# Indications, Dwell Time, and Removal Reasons of Standardized Mid-Thigh Lower-Extremity PICCs in Adult ICU Patients: A Retrospective Cohort Study

**DOI:** 10.3390/life16020262

**Published:** 2026-02-03

**Authors:** Wei-Hung Chang, Ting-Yu Hu, Hui-Fang Hsieh, Kuang-Hua Cheng, Kuan-Pen Yu

**Affiliations:** 1 Department of Critical Care Medicine, MacKay Memorial Hospital, Taipei 10449, Taiwanjeff01@mmh.org.tw (K.-H.C.); 2 Department of Nursing, MacKay Memorial Hospital, Taipei 10449, Taiwan

**Keywords:** lower-extremity PICC, intensive care unit, vascular access, ultrasound guidance, catheter dwell time, catheter removal, device stewardship, catheter-related infection, total parenteral nutrition, intravenous antibiotics

## Abstract

Lower-extremity peripherally inserted central catheters (PICCs) are used in critically ill adults when upper-extremity access is limited, yet real-world data on indications, dwell time, and device-related outcomes remain scarce. We retrospectively reviewed consecutive ultrasound-guided mid-thigh lower-extremity PICC placements performed under a standardized protocol (15 cm below the inguinal ligament; fixed 55-cm insertion depth) in an adult ICU and extracted indication patterns, catheter dwell time, removal reasons, and microbiological findings. Among 38 placements in 37 patients, difficult peripheral access was present in all cases; prolonged intravenous antibiotics were the predominant indication (34/38, 89.5%), followed by total parenteral nutrition (13/38, 34.2%) and vasopressor therapy (2/38, 5.3%). Median dwell time was 19.5 days (IQR 12–25; range 3–48). Catheters were most commonly removed due to death (15/38, 39.5%), discharge (13/38, 34.2%), or no longer being clinically indicated (8/38, 21.1%), while removal for suspected catheter infection/fever occurred in 2/38 (5.3%). A catheter-drawn culture was positive in 1/38 (2.6%; Candida albicans), whereas peripheral blood cultures were positive in 4/38 (10.5%). In this single-center retrospective descriptive cohort, standardized mid-thigh lower-extremity PICCs were used for prolonged venous access. Removals for suspected infection/fever evaluation were uncommon; however, CRBSI was not adjudicated and thrombosis surveillance was not performed. These findings describe local utilization patterns and support future comparative studies and stewardship-focused quality improvement.

## 1. Introduction

Central venous access is foundational to modern intensive care, enabling reliable administration of vasoactive agents, parenteral nutrition, blood products, and prolonged intravenous therapies when peripheral access is unstable or repeatedly fails [1]. Among available central venous access devices, peripherally inserted central catheters (PICCs) have become widely used because they can be placed at the bedside under ultrasound guidance, avoid some puncture-site complications of centrally inserted catheters, and support medium-to-long duration therapy without repeated peripheral cannulation [2,3]. However, PICCs are not low-maintenance devices: clinically meaningful complications—including catheter-related bloodstream infection (CRBSI), venous thrombosis, occlusion, and unplanned removal—remain important, particularly in critically ill populations with multi-organ dysfunction, frequent device manipulation, and high antimicrobial exposure [4,5,6]. These safety concerns have fueled the broader concept of vascular access “stewardship,” emphasizing appropriate device selection, minimizing unnecessary device-days, and ensuring timely removal once the clinical indication ends [7].

In adult ICU practice, PICCs are most commonly placed via upper-extremity veins; nevertheless, upper-extremity access is sometimes not feasible due to generalized edema, upper-limb burns or trauma, skin breakdown, limited venous reserve, concomitant devices, or the need to preserve veins for future access [8]. In such scenarios, lower-extremity PICC placement may function as a pragmatic alternative to secure durable venous access, especially when internal jugular or subclavian cannulation is undesirable or repeatedly unsuccessful [9]. Yet lower-extremity routes occupy a clinically cautious space: historical data linking femoral venous catheterization to higher infection and thrombosis risk compared with other insertion sites have shaped persistent hesitancy and guideline-level caution regarding lower-limb central access [10,11]. Meanwhile, contemporary bedside practice has evolved toward ultrasound-guided cannulation, standardized insertion protocols, and improved dressing/securement strategies, which may mitigate some risks traditionally attributed to femoral routes [12]. As a result, lower-extremity PICCs are increasingly used in select ICU cases, but real-world evidence remains limited and heterogeneous across institutions. In the ICU, central access is commonly achieved via centrally inserted catheters (e.g., internal jugular or subclavian) or femoral central venous catheters when rapid, high-acuity access is required. PICCs are generally considered when a longer treatment horizon is anticipated (e.g., prolonged intravenous antibiotics or parenteral nutrition), and appropriateness frameworks such as the Michigan Appropriateness Guide for Intravenous Catheters (MAGIC) emphasize aligning device selection with expected duration and clinical context. In patients in whom upper-extremity PICC placement is not feasible, a lower-extremity PICC may serve as an alternative to avoid repeated or undesirable central cannulation attempts.

Beyond the technical success of insertion, a practical—and frequently underreported—dimension is how these devices are actually used in ICU workflows: why they are inserted (indications), how long they remain in place (dwell time), and why they are removed (removal reasons). These utilization metrics are not merely administrative. Dwell time is a direct proxy for device-days, which is a key exposure variable for infection risk and a target for prevention programs [5,12]. Removal reasons (e.g., completion of therapy, transition of care, suspected infection, occlusion, or death) reflect both patient trajectories and the effectiveness of device necessity assessment processes [7,13]. However, utilization-focused data on lower-extremity PICCs in adult ICU practice—particularly indications, dwell time, and removal reasons—remain limited.

Lower-extremity PICCs introduce additional uncertainty regarding infection surveillance and device-related decision-making. ICU patients often develop fever and leukocytosis from non-catheter sources, prompting line evaluation and sometimes pre-emptive removal [5,6]. Conversely, critically ill patients may retain lines despite diminishing indications due to competing priorities and the perceived difficulty of re-establishing access if the line is removed prematurely [13]. Understanding how frequently lower-extremity PICCs are removed due to suspected infection (as opposed to end-of-life care, discharge, or resolution of indication) provides actionable insight into whether these devices are being used as “bridges” for definitive therapy, or instead accumulating low-value device-days that could be reduced through structured daily review [7,12,14]. From a hospital operations perspective, this matters: unnecessary central access can increase nursing workload, require additional imaging and dressing changes, and increase the downstream burden of blood culture collection and antimicrobial escalation when infection is suspected [5,13].

Accordingly, using a standardized mid-thigh ultrasound-guided lower-extremity PICC placement protocol in an adult ICU, we conducted a retrospective cohort study to characterize (i) the clinical indications for lower-extremity PICC insertion, (ii) catheter dwell time, (iii) reasons for catheter removal, and (iv) microbiological findings temporally associated with PICC use. By focusing on utilization and stewardship outcomes—rather than catheter tip positioning or malposition risk—we aim to provide a complementary, practice-facing evidence layer for centers that employ lower-extremity PICCs when upper-extremity access is not feasible [9,10,12].

## 2. Materials and Methods

### 2.1. Study Design and Setting

This retrospective cohort study was conducted in the adult intensive care unit (ICU) of MacKay Memorial Hospital, Taipei, Taiwan. We included consecutive standardized mid-thigh lower-extremity PICC placements performed between 29 August 2022, and 1 April 2025, as documented in the electronic medical record. The unit of analysis was each PICC placement.

### 2.2. Participants and Eligibility Criteria

We included adult ICU patients who underwent standardized mid-thigh lower-extremity PICC placement and had complete documentation sufficient to determine: (i) clinical indication(s) for insertion, (ii) catheter dwell time, and (iii) catheter removal reason. Records were excluded if key variables required for the primary analyses (insertion/removal dates, indication, or removal reason) were missing.

Because one patient underwent more than one lower-extremity PICC placement, the primary analysis treated each placement as an independent observation, and a sensitivity analysis (prespecified) was performed using only the first placement per patient to assess robustness.

### 2.3. Standardized Lower-Extremity PICC Placement Protocol and Procedural Variables

**Figure 1:**
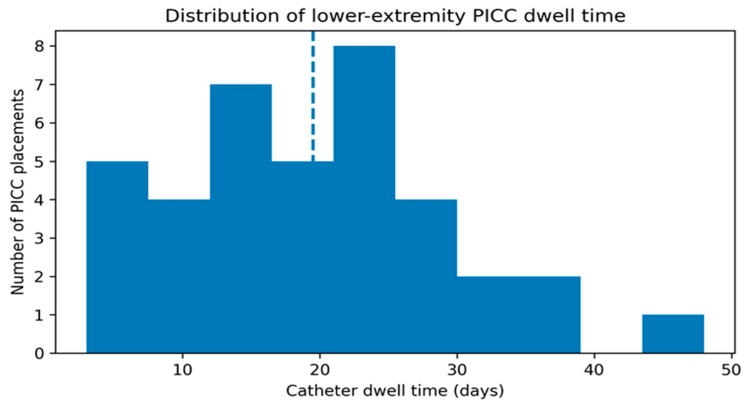
Histogram of catheter dwell time (days) for standardized lower-extremity PICC placements. The dashed line indicates the median dwell time.

All PICCs were placed using a standardized mid-thigh approach. The puncture point was defined as 15 cm below the inguinal ligament. Catheters were advanced to a standardized insertion depth of 55 cm as part of an institutional protocol intended to (i) reduce operator-dependent variation, and (ii) provide a consistent workflow for bedside placement when radiographic tip positioning was not the focus of this utilization study. Because this study focused on utilization rather than tip position, the standardized insertion depth was applied as a workflow parameter and was not intended to serve as a validated surrogate for central catheter tip location. This study did not aim to validate optimal tip location. A schematic of the standardized puncture site and catheter advancement pathway is provided in Figure 1.

For each placement, the following procedure-related variables were recorded from procedural documentation: insertion laterality (left vs. right lower extremity); femoral artery–vein relationship pattern at the puncture site; and device descriptors, including the number of lumens (single- vs. dual-lumen) and manufacturer/brand.

In our institution, “insertion depth” refers to the length advanced from the puncture site (standardized at 55 cm per protocol), whereas device labels documented in the record (e.g., “40 cm”) refer to catheter product specifications. These represent distinct data fields and were not used to infer or adjudicate catheter tip location.

### 2.4. Outcomes and Operational Definitions

#### 2.4.1. Operational Definition of Indications for Lower-Extremity PICC Placement

Indications were abstracted from the documented placement rationale and categorized based on bedside clinical use into the following (non-mutually exclusive) indication groups:

- Difficult peripheral intravenous access;
- Prolonged intravenous antibiotics;
- Total parenteral nutrition (TPN);
- Vasopressor therapy (e.g., norepinephrine/Levophed).

Because this was a retrospective utilization study, detailed physiologic severity scores (e.g., APACHE II or SOFA), bedside stability assessments, and whether the placement was elective versus emergent were not reliably available in a standardized format across all records. As a pragmatic proxy for concurrent high-acuity therapy at the time of placement, we extracted whether vasopressor therapy and/or TPN and/or prolonged intravenous antibiotics were documented as indications (non-mutually exclusive).

#### 2.4.2. Catheter Dwell Time

**Dwell time** was defined as the number of days from the PICC insertion date to the PICC removal date, as recorded in the clinical record. When available, admission and discharge dates were also recorded to contextualize catheter use within the ICU/hospital course.

#### 2.4.3. Reasons for Catheter Removal

Removal reasons were extracted from clinical documentation and categorized a priori into mutually exclusive operational groups:

- Death: catheter removal in the context of in-hospital death.
- Discharge/transfer: catheter removal performed as part of a transition-of-care process (ICU-to-ward transfer, inter-hospital transfer, or hospital discharge), when documentation indicated no immediate device-related complication prompting removal.
- No longer clinically indicated: documentation that the original indication had resolved or that the device was no longer required for ongoing therapy. This category included (i) completion or cessation of intravenous antibiotics, (ii) discontinuation of TPN, and/or (iii) establishment of alternative vascular access making the lower-extremity PICC unnecessary (e.g., upper-extremity access became feasible or a CVC was placed), as explicitly stated in notes.
- Suspected infection/fever evaluation: catheter removed because of suspected catheter-related infection or unexplained fever leading to line evaluation, as documented by the treating team.

If multiple reasons were documented, the primary reason driving removal (as stated in the clinical note) was used.

#### 2.4.4. Microbiological Findings During PICC Use

Microbiological results were abstracted when blood cultures were obtained during catheter use or in the context of line evaluation. Two sources were recorded separately:

- Catheter-drawn (PICC) blood culture results;
- Peripheral blood culture results.

A culture was considered **positive** if any organism was reported as grown in the final culture result.

### 2.5. Data Collection and Variables

Data were extracted from electronic medical records and procedural documentation. Variables included: age, sex, height, admission weight, dosing weight, primary clinical diagnosis category at ICU admission, insertion laterality, femoral artery–vein relationship pattern, device descriptors (type/brand), insertion and removal dates, dwell time, removal reason, microbiology results (PICC and peripheral), and final disposition (e.g., discharged, died, or transferred).

### 2.6. Statistical Analysis

Continuous variables are summarized as **median (interquartile range, IQR)** and categorical variables as **count (percentage)**. The primary analyses were descriptive, focusing on: (i) indication patterns, (ii) dwell time distribution, (iii) removal reason distribution, and (iv) culture positivity rates from PICC and peripheral sources.

A sensitivity analysis was performed restricting the dataset to the first placement per patient to evaluate whether repeated placement from a single patient materially influenced descriptive estimates. Analyses were performed using Python (version 3.10) with the pandas and SciPy libraries.

### 2.7. Ethics Approval

The study was conducted in accordance with the Declaration of Helsinki and approved by the Institutional Review Board of MacKay Memorial Hospital (IRB No. 21MMHIS430e, date of approval 14 January 2022). The requirement for informed consent was waived due to the retrospective design and use of de-identified data.

### 2.8. Data Availability

Individual-level clinical data contain potentially identifiable information and are not publicly available. De-identified data supporting the findings of this study may be made available by the corresponding author upon reasonable request and subject to institutional and ethical approval.

## 3. Results

A total of 38 standardized mid-thigh lower-extremity PICC placements in 37 patients were included.

### 3.1. Baseline and Procedural Characteristics

Baseline patient characteristics and procedural features of the 38 lower-extremity PICC placements (37 patients) are summarized in Table 1.

### 3.2. Indications for Lower-Extremity PICC Placement

Difficult peripheral intravenous access was cited in all placements (38/38, 100%). Prolonged intravenous antibiotic therapy was the most common indication (34/38, 89.5%), followed by total parenteral nutrition (TPN) (13/38, 34.2%) and vasopressor therapy (2/38, 5.3%). These patterns reflect the predominant use of lower-extremity PICCs as a durable access strategy when upper-extremity venous access was limited and ongoing ICU therapies required reliable central access.

Indications were non-mutually exclusive; 13/38 placements involved TPN and 2/38 involved vasopressor therapy, indicating concurrent high-acuity infusion needs in a subset of placements (Table 2).

Overall catheter dwell time was a median of 19.5 days (IQR 12–25), with a range of 3–48 days (Figure 1). Across the study period, dwell time distribution was right-skewed, with most devices remaining in place for approximately 1–4 weeks and a smaller number extending beyond 30 days. When stratified by removal reason, dwell time tended to be shorter among catheters removed in the context of death (median 12.0 days) and longer among catheters removed because they were no longer clinically indicated (median 24.0 days) (Table 3).

**Figure 2:**
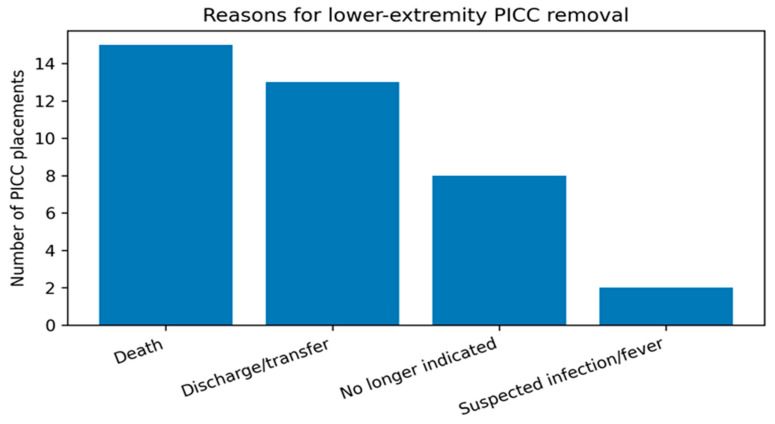
Documented reasons for lower-extremity PICC removal (counts). Categories are mutually exclusive as recorded in the clinical record.

Reasons for catheter removal are summarized in Table 3 and shown in Figure 2. The most frequent documented removal reason was death (15/38, 39.5%), followed by discharge/transfer (13/38, 34.2%) and removal due to no longer being clinically indicated (8/38, 21.1%). Removal triggered by suspected catheter-related infection or fever evaluation was uncommon (2/38, 5.3%). Taken together, these data suggest that the majority of lower-extremity PICCs were discontinued because of patient disposition or overall clinical trajectory rather than device complications prompting urgent removal.

Microbiological results obtained during catheter use or in the context of line evaluation were recorded separately for catheter-drawn and peripheral blood cultures. A positive catheter-drawn (PICC) culture was documented in 1/38 placement (2.6%; Candida albicans). Peripheral blood cultures were positive in 4/38 placements (10.5%; organisms included Candida albicans, carbapenem-resistant Acinetobacter baumannii (CRAB), carbapenem-resistant Klebsiella pneumoniae (CRKP), and Bacillus species). Because the dataset was not designed to adjudicate catheter-related bloodstream infection using standardized clinical criteria, these microbiological findings are reported descriptively to contextualize line evaluation and removal decisions. A summary of culture sources, sampling frequency, positivity, and organisms identified is provided in Table 4.

**Figure 3:**
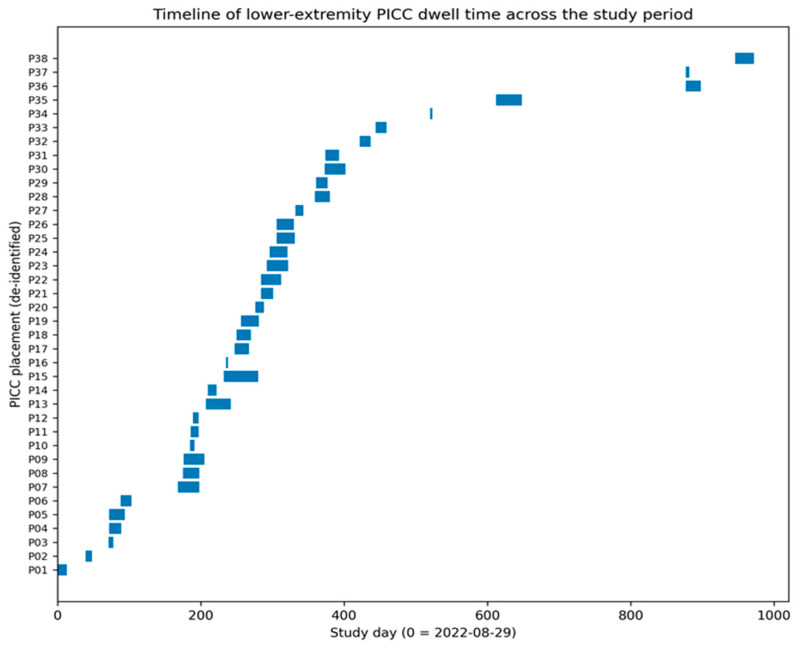
Timeline of lower-extremity PICC dwell time across the study period. Each horizontal bar represents one PICC placement (de-identified) from insertion to removal; the *x*-axis is shown as study day relative to the first placement date.

A prespecified sensitivity analysis restricted to the first placement per patient (*n* = 37) yielded similar descriptive estimates, with a median dwell time of 19.0 days and a comparable distribution of removal reasons. The timeline of lower-extremity PICC dwell time across the study period is shown in Figure 3.

## 4. Discussion

In this cohort, lower-extremity PICCs were predominantly used to support prolonged intravenous therapy with extended dwell times, and most devices were removed due to patient disposition or end-of-life outcomes rather than suspected catheter infection, highlighting the potential value of structured daily device-necessity assessment to optimize lower-extremity PICC use in ICU practice [7,12,14,15].

This retrospective cohort study describes how standardized mid-thigh lower-extremity PICCs were actually used in an adult ICU—why they were inserted, how long they remained in place, and why they were removed. Thrombosis surveillance was not performed; therefore, no inference regarding thrombotic safety can be made. The main operational message is simple: in a setting where upper-extremity access is often not feasible, lower-extremity PICCs with a supported prolonged intravenous therapy with a median dwell time of 19.5 days, and most devices were removed due to patient trajectory (death or discharge) or resolution of clinical need rather than suspected catheter infection. From a stewardship and workflow standpoint, these findings matter because they separate two very different problems: (i) complications that mandate urgent removal, and (ii) “low-value device-days” that accumulate when ongoing indication is not systematically reassessed [16,17].

### 4.1. Indications: Lower-Extremity PICCs as a Pragmatic ICU Access Strategy

In this cohort, difficult peripheral access was present in essentially all cases, and prolonged IV antibiotic therapy was the predominant documented indication. This aligns with the practical reality that ICU vascular access decisions are frequently constrained by anatomy and urgency rather than preference: once peripheral access is unreliable, clinicians default to devices that can deliver prolonged therapy safely and predictably, especially when clinical trajectories are uncertain [18,19,20]. From a systems perspective, lower-extremity PICCs in this context function as a “continuity-of-therapy asset”—they reduce repeated cannulation attempts and limit treatment interruptions due to peripheral IV failure, which is an underappreciated driver of nursing workload and therapy delays [18,19]. At the same time, the high prevalence of antibiotic-driven placement highlights the importance of appropriateness thinking: the catheter is not the goal; uninterrupted, safe therapy is the goal [20].

### 4.2. Dwell Time: A Useful Exposure Metric, Not Just a Calendar Number

The observed dwell time distribution suggests that lower-extremity PICCs were used for sustained therapy rather than short, bridging access. In published ICU practice surveys, PICC dwell time is often shorter (median around ~9 days), and most centers report median dwell times ≤ 14 days [16,19]. In contrast, the median dwell time observed in our cohort was 19.5 days, suggesting that lower-extremity PICCs in this setting were used in a longer-horizon treatment context. This utilization pattern is directionally consistent with appropriateness frameworks (e.g., MAGIC), which emphasize aligning PICC use with expected duration and clinical context. Dwell time is clinically relevant because it approximates device-days—an exposure variable linked to infection surveillance, catheter maintenance burden, and cumulative complication risk [21,22,23]. ICU workflows sometimes unintentionally promote prolonged device retention: removing a functioning central line can feel risky when re-access may be difficult later. That risk-aversion is understandable, but it can create a predictable trade-off: fewer emergency line insertions at the cost of more device-days [21,22]. The appropriate management response is not “remove early at all costs,” but rather “remove promptly when the indication is no longer defensible,” supported by standardized review processes and clear decision thresholds [23].

### 4.3. Removal Reasons: The Highest-ROI Target for Improvement Is Often Process, Not Technology

Most removals in this cohort occurred due to death, discharge/transfer, or documentation that the device was no longer clinically indicated, whereas removals for fever/infection evaluation were infrequent. Interpreted descriptively, removals for **suspected infection/fever evaluation** were uncommon in this cohort; however, this represents a documented **removal decision/process** rather than an adjudicated infection outcome [24,25]. In other words, the catheter was often “doing its job,” but the organization still pays an operational tax if the device remains in place by default rather than by active decision-making [25].

This is where the ROI is highest: implementing a structured daily device-necessity checkpoint (e.g., ICU line rounds, checklist prompts in progress notes, or nursing-driven escalation when indications lapse) is usually cheaper and more reliable than adding new devices or escalating complexity [26,27,28]. These stewardship processes are conceptually similar to other ICU device-reduction initiatives: success comes from making the right action (remove when unnecessary) the path of least resistance [26,27]. Importantly, “no longer indicated” removals should not be framed as failure; they are evidence that device-days can be actively managed. The risk is not that the line was removed, but that it may have been retained longer than needed because no one owned the decision [28].

### 4.4. Microbiological Findings: Signal, Noise, and the ICU Fever Problem

Only a small fraction of catheter-drawn cultures were positive, while peripheral cultures were more frequently positive. This pattern is not unexpected in the ICU, where fever prompts broad diagnostic workups and bloodstream infections may originate from non-catheter sources (lungs, abdomen, urinary tract, skin/soft tissue) [29,30]. Two cautions are warranted. First, culture collection is not standardized in retrospective cohorts; sampling bias is unavoidable (cultures are obtained when clinicians suspect infection). A low frequency of removals for suspected infection does not equate to a low incidence of catheter-related infection in ICU patients, where competing infectious sources are common and culture sampling was not standardized [30]. Therefore, our data are best interpreted as utilization-facing microbiological context rather than a definitive CRBSI rate estimate [30].

These observations are descriptive and hypothesis-generating; comparative studies with standardized outcome definitions are required before any safety inference [30].

### 4.5. Clinical Implications: What This Means for ICU Teams Tomorrow Morning

The practical implications are straightforward and implementable without new equipment. In our cohort, 8/38 (21.1%) catheters were removed because they were no longer clinically indicated, supporting daily device-necessity assessment as a concrete stewardship target:

1. **Clarify indication at insertion and codify it in the chart.** Indication drift is common; explicit documentation supports later de-escalation decisions [31].
2. **Institutionalize a daily “line necessity” check.** If a line is kept “just in case,” that should be a conscious decision with explicit rationale, not a default state [31,32].
3. **Separate “fever evaluation” from “line removal reflex.”** In a high-noise ICU environment, removing the line at the first temperature spike may not be the most efficient risk-management strategy; structured evaluation pathways reduce unnecessary removals and reinsertions while maintaining safety [32,33].

These implications align with appropriateness frameworks that emphasize choosing the right device for the right indication and minimizing avoidable device-days—especially when devices become embedded in complex care pathways (Box 1) [33].

Box 1 **Daily lower-extremity PICC necessity checklist (example).**

(1) Indication today (mandatory): Abx/TPN/vasoactive infusion/poor access/other
(2) Expected remaining duration: ≤3 days/4–7 days/>7 days
(3) De-escalation options: switch to oral therapy; stop TPN; alternative access available (e.g., upper-extremity access feasible); remove if none apply
(4) If no clear indication: assign owner (attending/on-call) and set removal target within 24 h

### 4.6. Limitations

This analysis has predictable constraints. It is a single-center retrospective cohort with a modest sample size, and it does not include a comparator group (e.g., upper-extremity PICCs or centrally inserted catheters) to support comparative effectiveness claims [34]. Culture acquisition and documentation practices were not standardized, limiting causal attribution for microbiological findings. Thrombotic outcomes were not captured using protocolized ultrasound screening; asymptomatic lower-extremity DVT may therefore have been missed, and no inference regarding thrombotic safety can be made [34]. Finally, removal reasons are derived from clinical documentation and may not fully reflect the decision-making dynamics at the bedside. We did not systematically capture the specific bedside reasons for choosing a lower-extremity route (e.g., burns, trauma, obesity, or upper-extremity device constraints), which limits contextual interpretation of device-selection decisions.

### 4.7. Future Directions

Future work should prioritize multi-center validation with larger samples and standardized definitions for catheter-related infection outcomes and thrombosis surveillance [35]. Prospective designs could evaluate whether simple stewardship interventions (e.g., daily indication prompts, removal triggers, or device selection pathways) reduce unnecessary device-days without increasing urgent reinsertions or therapy interruptions [35]. Additionally, integrating process measures (time-to-use, number of access attempts avoided, imaging requirements, and nursing workload) would allow a more complete assessment of clinical value beyond complication rates alone.

In summary, this single-center retrospective descriptive cohort characterizes indications, dwell time, and removal reasons for standardized mid-thigh lower-extremity PICCs in adult ICU practice. Removals for suspected infection/fever evaluation were uncommon in this cohort, but these findings are descriptive and require comparative validation with standardized infection and thrombosis outcome definitions [16,17,35].

## 5. Conclusions

In this adult ICU cohort, standardized mid-thigh lower-extremity PICCs were predominantly used to support prolonged intravenous therapy in patients with difficult peripheral access, with a median dwell time of 19.5 days and removals driven mainly by patient trajectory (death or discharge/transfer) or resolution of clinical indication rather than infection-triggered evaluation. These findings describe local utilization patterns under a standardized workflow; comparative studies with standardized infection and thrombosis definitions are required before any safety inference.

## Figures and Tables

**Table 1:** Baseline patient characteristics and procedural features of standardized lower-extremity PICC placements (*n* = 38 placements, 37 patients).

Variable	Value
**Age, years**, median (IQR)	73 (69–83.75)
**Sex**, *n* (%)	
— Male	26 (70.3)
— Female	11 (29.7)
**Laterality of placement**, *n* (%)	
— Right lower extremity	26 (68.4)
— Left lower extremity	12 (31.6)
**Femoral artery–vein relationship**, *n* (%)	
— Artery–vein parallel	30 (78.9)
— Artery on vein	8 (21.1)
**Catheter characteristics**, *n* (%)	
— Dual-lumen PICC	38 (100)
— Manufacturer: Arrow	34 (89.5)
— Manufacturer: BD	4 (10.5)
**Catheter dwell time, days**, median (IQR)	19.5 (12–25)
**Catheter dwell time, range**, days	3–48
**Study period**	29 August 2022–1 April 2025

Values are reported per PICC placement unless otherwise specified.

**Table 2:** Indications for standardized lower-extremity PICC placement.

Indication (Non-Mutually Exclusive)	*n*	%
Difficult peripheral intravenous access	38	100.0
Prolonged intravenous antibiotics	34	89.5
Total parenteral nutrition (TPN)	13	34.2
Vasopressor therapy	2	5.3

Data are shown as count (percentage). Indications were extracted from procedure documentation and were not mutually exclusive.

**Table 3:** Catheter dwell time and reasons for lower-extremity PICC removal.

Removal Reason	*n*	%	Dwell Time, Days (Median [IQR])
Death	15	39.5	12.0 [7.5–25.0]
Discharge/transfer	13	34.2	20.0 [15.0–24.0]
No longer indicated	8	21.1	24.0 [21.8–27.0]
Suspected infection/fever	2	5.3	14.5 [13.8–15.2]

Overall dwell time: median 19.5 days (IQR 12–25); range 3–48 days.

**Table 4:** Summary of microbiological cultures obtained during lower-extremity PICC use.

Culture Source	No. of Episodes Sampled (N)	Positive, *n*/N (%)	Organisms Identified (Positive Episodes)
Catheter-drawn (PICC) blood culture	38 *	1/38 (2.6%)	*Candida albicans* (*n* = 1)
Peripheral blood culture	38 *	4/38 (10.5%)	*Candida albicans* (*n* = 1); CRAB (*n* = 1); CRKP (*n* = 1); *Bacillus* species (*n* = 1)

* Culture data were abstracted from routinely documented clinical records. For each PICC placement, culture results were recorded as organism identified or “no growth” in the database. The dataset does not distinguish between cultures that were not obtained and cultures that were obtained with negative results, nor does it capture the number or timing of repeated cultures during a single PICC dwell period. Therefore, N reflects the number of placements with recorded culture fields rather than protocolized sampling frequency.

## Data Availability

The data presented in this study are not publicly available due to ethical and privacy restrictions. De-identified data supporting the findings of this study are available from the corresponding author upon reasonable request and subject to institutional approval.

## Abbreviations

PICC, Peripherally Inserted Central Catheter; ICU, Intensive Care Unit; IV, Intravenous; TPN, Total Parenteral Nutrition; CRBSI, Catheter-Related Bloodstream Infection; IQR, Interquartile Range; IRB, Institutional Review Board.
